# Superhydrophobic magnetic sorbent via surface modification of banded iron formation for oily water treatment

**DOI:** 10.1038/s41598-022-15187-6

**Published:** 2022-06-30

**Authors:** Mohsen Farahat, Ahmed Sobhy, Moustafa M. S. Sanad

**Affiliations:** Central Metallurgical Research and Development Institute, Helwan, 11421 Cairo Egypt

**Keywords:** Environmental sciences, Chemical engineering

## Abstract

In the current study, a simple dry coating method was utilized to fabricate a super-hydrophobic super-magnetic powder (ZS@BIF) for oily water purification using zinc stearate (ZS) and banded iron formation (BIF). The produced composite was fully characterized as a magnetic sorbent for oily water treatment. The results of X-ray diffraction diffractometer (XRD), Fourier transform infrared (FTIR), X-ray photoelectron spectroscopy (XPS), scanning electron microscope (SEM), energy-dispersive X-ray spectroscopy (EDS) and particle size analysis revealed the fabrication of homogenous hydrophobic-magnetic composite particles with core–shell structure. Contact angle and magnetic susceptibility results showed that 4 (BIF): 1 (Zs) was the ideal coverage ratio to render the core material superhydrophobic and preserve its ferromagnetic nature. The capability of the fabricated composite to sorb. *n*-butyl acetate, kerosene, and cyclohexane from oil–water system was evaluated. ZS@BIF composite showed a higher affinity to adsorb cyclohexane than n-butyl acetate and kerosene with a maximum adsorption capacity of about 22 g g^−1^ and 99.9% removal efficiency. Moreover, about 95% of the adsorbed oils could be successfully recovered (desorbed) by rotary evaporator and the regenerated ZS@BIF composite showed high recyclability over ten repeated cycles.

## Introduction

Oil spills occurred due to natural disasters, human activities, or industrial mistakes, damage water resources and cause high environmental risks. This kind of pollution attracted the attention of the research community to develop several procedures for pollution control and oil spill recovery^1–4^. For instance, when oil spills formed, a thin layer of oil covers a high area of water surface, which destroys marine life^5^. Besides, oil spill control and recovery are costly and many cleaning techniques do not permit complete oil recovery from water^5^. Various oil/water separation routes include booms, skimmers, sorbents, dispersants, in situ burning, bioremediation, and magnetic nanocomposites^6–9^. The new trends in oil/water separation consider some essential points including surface wettability (hydrophobicity degree), porosity, and roughness of numerous filtration/absorption surfaces. The separation materials with modified surfaces are usually fabricated in different shapes such as meshes^10,11^, textiles^12–14^, foams^15,16^, sponges^17–19^, films^20^, and membranes^21^. Honestly, most of the employed materials in the previous methods such as polypropylene, hydrazine hydrate, and graphene oxide are synthetic, expensive, harmful, and hard to biodegrade^22^. Recently, huge efforts have been paid to nature-derived materials such as marine sponges modified with vegetable waxes^23^ to eliminate some of these drawbacks. In addition, the combination of such hydrophobic surfaces with magnetic materials has attracted much attention due to the facile and fast collection of the oil-loaded materials from the aqueous medium at the end of the separation step. In this regard, magnetite nanoparticles coated with silica and (3-aminopropyl)triethoxysilane (APTS) illustrated a high efficiency for oily water treatment^24^. In addition, magnetite nanoparticles coated with cheap polyvinylpyrrolidone (PVP) prepared using a modified polyol method were used for the oil separation from water^25^.

On the other hand, recently, superhydrophobic sorbents have received more consideration in many applications, and the majority of surface modification methods suffer from many drawbacks^26^. These drawbacks include complex fabrication methods, weak stability in a severe environment, high energy consumption, and low adaptability^27^. Accordingly, the demand for fabricating new superhydrophobic sorbents with strong stability, simple production procedure, and high adaptability is urgent right now. Recently, zinc stearate^28^ and magnesium stearate^29^ have been utilized to coat sponges for oil/water treatment. Moreover, zinc stearate coated polyurethane sponges illustrated an adsorption capacity range of 6–81 g g^−1^ depending on the density of the sponge. Seth et al.^30^ coated cotton fabric with zirconium zinc stearate, which was highly efficient for oil/water separation. Tao et al.^31^ soaked a magnetic polyurethane sponge in a stearic acid solution to generate superhydrophobic characteristics, which was able to absorb oil from water with an absorption capacity of 16–60 g g^−1^. Previously, Tran and Lee^32^ treated polyurethane sponge with zinc oxide, stearic acid, and iron oxide to produce a superhydrophobic magnetic sponge applicable for oil/water separation. Xia et al.^33^ prepared a new magnetic foam from iron/iron carbide nanoparticles (Fe/Fe_3_C NPs) and polydimethylsiloxane (PDMS) coating, via ferric nitrate assisted chemical blowing and carbonization of polyvinylpyrrolidone (PVP). This developed showed high selectivity towards oil removal from water in non-open channels under assistance of a magnet. Polyurethane sponge loaded with magnetite nanoparticles also showed good superhydrophobic character for de-emulsification of toluene/water emulsions with excellent stability against sever acidic, basic, saline, cold and hot conditions^34^. Recently, natural iron oxide minerals have been used as a source of magnetism to develop magnetic sorbents for wastewater treatment applications^35,36^.

The present work is devoted to developing superhydrophobic magnetic composite via a simple-preparation technique called the dry coating process. It is summarized by using a magnetic iron ore such as banded iron formation (BIF) ore for the first time that can be hydrophobized with zinc stearate, which could be considered a cheap and efficient sorbent in oil spills removal due to its hydrophobicity and magnetic approachability.

## Materials and methods

### Materials

A representative banded iron formation (BIF) was collected from the Wadi Karim and Um Anab areas in the central Eastern Desert, the Red Sea region of Egypt. At the lab, the ore was crushed, ground, mixed, split into smaller portions, and sealed for later usage.

Cyclohexane (C_6_H_12_) with 99% purity, Kerosene of pure chemical grade, n-butyl acetate (CH_3_·COO·(CH_2_)_3_·CH_3_) with 98.5 purity, and zinc stearate (ZS) powder with 99.5% purity used in this study were supplied from El-Nasr Chemical Company.

### Synthesis of superhydrophobic magnetic sorbent

The representative sample of Egyptian Red Sea iron oxide ore (BIF) was utilized as the magnetic core for the sorbent material. The iron oxide content is about 70% with an average particle size of about 10 µm. The chemical grade zinc stearate (ZS) powder of 2 µm average particle size was implemented as a hydrophobic material. The samples of superhydrophobic magnetic sorbent (ZS@BIF) were prepared by loading two different amounts of ZS (1 and 5 g) on the surface of BIF particles achieving 5% and 20% weight ratio of ZS addition. The homogeneous mixing of both components was primarily carried out in the Ordered Mixture (O.M.) Dizer unit for 10 min. Each mixture was then moved to the hybridizer system Supplementary Fig. S1 (supplementary materials) (Nara Machinery Co. Ltd., Model: NHS-0, Tokyo, Japan). The coating of BIF particles was performed with the aid of Van der Waals forces as a result of the difference in the particle size. The high-speed rotation (12,000 rpm) operated through compressed air stream pumps leads to the generation of friction forces which causes the attraction of small particles on the surface of large ones forming a hydrophobic shell of ZS on the BIF core particles. The obtained magnetic sorbent samples ZS@BIF with two ZS contents (5% and 20%) were collected within 15 min of coting time.

### Characterization

#### Physicochemical characterization

The mineralogical composition of the iron ore sample and zinc stearate@banded iron formation composite (ZS@BIF) was characterized qualitatively using an X-ray diffraction diffractometer (Bruker D8 XRD). Fourier transform infrared (FTIR) (Spectrometer JASCO, 6300, Japan) was used for surface examination. The surfaces of the banded iron formation host and the fabricated composite ZS@BIF were deeply analyzed by X-ray photoelectron spectroscopy (XPS) using K-alpha Thermo Scientific AXIS 165 (Thermo Fisher Scientific, USA) spectrometer. The adsorption characteristics of n. butyl acetate (BA) and cyclohexane (CH) on ZS@BIF composites were evaluated using FTIR. The surface morphology investigations were performed using a scanning electron microscope (SEM), and the elemental mapping of zinc, iron and oxygen was observed by conducting Energy Dispersive X-Ray Spectroscopy EDS. Grain size analysis was conducted by laser particle size analyzer (BT-2001). Thermo Scientific Automatic pycnometer Pycnomatic ATC was used for density measurement.

#### Contact angle measurements

To investigate the hydrophobicity strength of the samples, the wettability of BIF and ZS@BIF composites with 5% and 20% ZS was estimated with JC2000A contact angle apparatus using the free sessile drop method. The smooth surface of the sample powder was prepared on a glass plate for the estimation of the contact angle. Then, a stable water droplet of about 1 mm diameter was placed on the sample surface using a microsyringe.

#### Magnetic susceptibility

High-resolution magnetic susceptibility readings from powder samples including BIF, 5% ZS@BIF, and 20% ZS@BIF were measured to evaluate their magnetic properties using MS2G meter manufactured by Bartington Instruments, Oxford, England. MS2G is designed for magnetic susceptibility measurements of powder samples.

### Performance evaluation

The absorption capacities of BIF, 5% ZS@BIF, and 20% ZS@BIF composites powder were studied to show the capability of the composites for the efficient removal of different kinds of oils from water. A 0.5 g of composite powder is placed between two layers of cotton on a funnel, and the oily water containing a defined volume of kerosene, butyl acetate, or cyclohexane is allowed to flow through the composite powder to a conical flask. After complete and saturated adsorption, the volume of un-adsorbed oil was measured and then the adsorption capacity *Q* in g g^−1^ was calculated as follow:

$$Q = \, \left( {Ci - Ce} \right)/m,$$

where *Ci* is the initial mass of oil in g, *Ce* is the mass of oil at equilibrium, and *m* is the mass of composite in g.

The advantages of the magnetic and hydrophobic abilities of the superhydrophobic composites were investigated. The oil was poured on the water surface, then the composite particles were spread in the oil/water mixture, and oil is absorbed into the particles quickly forming the particles/oil layer. The particle/oil phase was then removed by applying an external magnetic field. In addition, a rotary evaporator was used to distill the adsorbed oil off and recover the adsorbent for multi-cycle reuse.

## Results and discussion

### Physicochemical characterization

**Figure 1:**
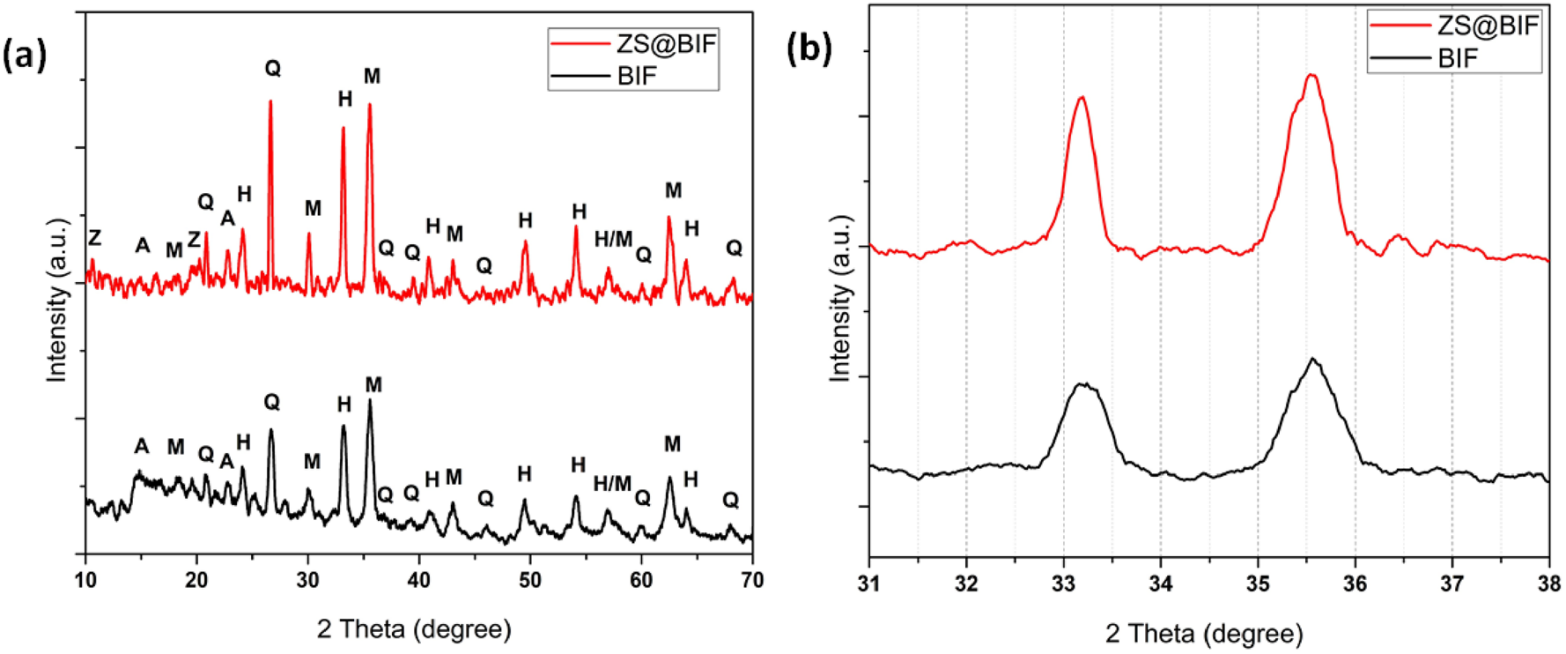
(**a**) XRD patterns of banded iron formation (BIF) and zinc stearate@banded iron formation composite (ZS@BIF), (**b**) Shift of XRD peaks to lower angles. [Albite (A), Hematite (H), Magnetite (M), Quartz (Q), Zinc stearate (Z)].

The XRD pattern results of BIF and ZS@BIF (20%ZS) are given in Fig. 1a,b, which indicate that the major minerals are magnetite (Fe_3_O_4_), hematite (Fe_2_O_3_), quartz (SiO_2_), and albite (NaAlSi_3_O_8_) in BIF, while ZS@BIF has the same mineralogy in addition to zinc stearate peaks with a slight shift in the XRD peaks to a lower angle.

**Figure 2:**
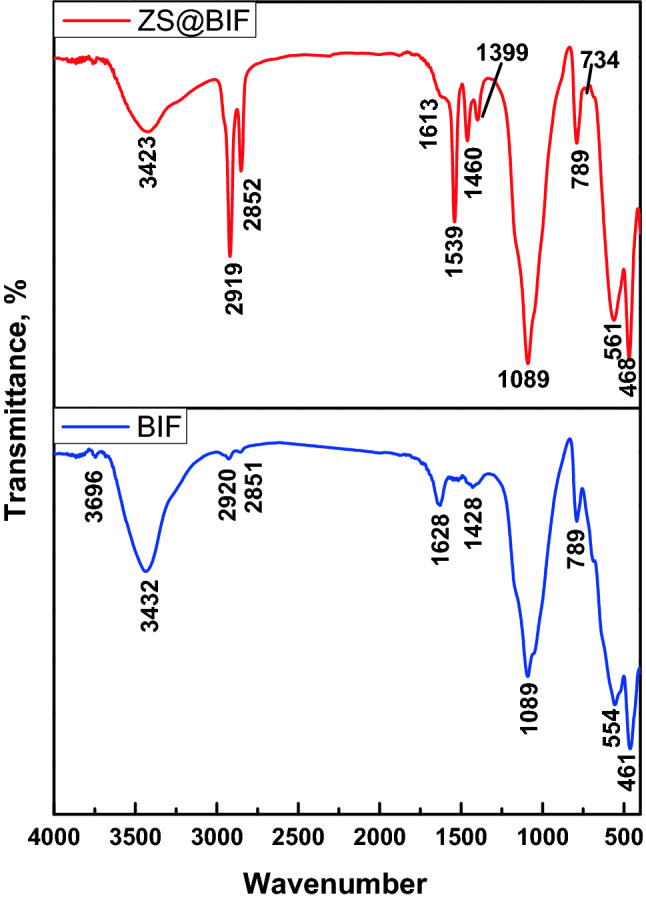
FTIR spectra of BIF and 20% ZS@BIF composite.

The FTIR patterns of BIF and ZS@BIF composite are presented in Fig. 2. In the BIF spectrum, the observed peak at 3432 cm^−1^ is assigned to the vibration and stretching of hydroxyl bonds (OH) of the absorbed water^37,38^. The bands at 2851 cm^−1^ and 2920 cm^−1^ are ascribed to the asymmetric and symmetric C–H bonds^39,40^. The observed band at 1628 cm^−1^ in the BIF spectrum is attributed to the physically adsorbed H–O–H bonds. The two bands observed at 1089 cm^−1^ and 789 cm^−1^ are assigned to the bending vibrations of Si–O–Si bonds^41^. The two absorption bands at 554 cm^−1^ and 461 cm^−1^ are related to the F–O bond's bending mode existing in the two crystallines α-Fe_2_O_3_ and Fe_3_O_4_^42^.

The characteristic peaks of zinc stearate were observed in the FTIR spectrum of ZS@BIF in addition to the observed peaks of BIF. In particular, the peaks of symmetric and antisymmetric COO– bands were observed at 1398 cm^−1^ and 1539 cm^−1^ respectively^42^. The peaks observed at 2919, 2852, and 1460 cm^−1^ are attributed to symmetric and antisymmetric H–C–H bonds^42^. Moreover, the characteristic peak of the Zn–O chelating bond could be observed at 1539 cm^−1^^42^.

**Figure 3:**
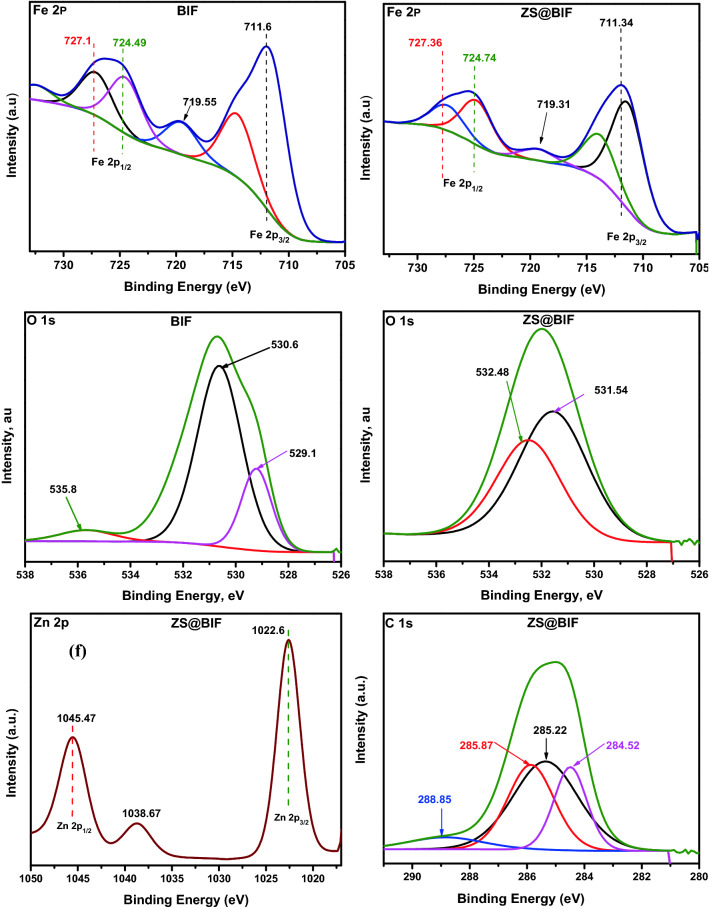
XPS spectra for BIF and ZS@BIF samples.

Fe2p, O1s high-resolution XPS spectra for BIF and ZS@BIF samples in addition to Zn2p and C1s spectra for ZS@BIF composite are shown in Fig. 3. As in Fig. 3a,b, the Fe2p spectra for both samples are identical where the two main peaks of Fe2p3/2 and Fe2p1/2 were positioned around 711 eV and 725 eV, respectively which is in agreement with those observed for hematite α-Fe_2_O_3_ and magnetite Fe_3_O_4_ phases confirming the results of XRD investigation^40–43^.

The O1s spectra for BIF and ZS@BIF are shown in Fig. 3c,d. For the BIF sample, the observed peaks at 529.1 and 530.6 eV are attributed to Fe–O bonds^44^, and the peak observed at 535.8 eV is due to the O–H bond of adsorbed water^45,46^. Conversely, in the O1s spectrum of ZS@BIF, as shown in Fig. 3d, the characteristic peak of Zn–O was observed at binding energy 531.54^47^ while the peak was observed at 538.48 eV may be attributed to the aliphatic C=O bond in zinc stearate^48^. It is noteworthy that the absence of O–H characterized peak of adsorbed water in O1s in the ZS@BIF sample is due to the superhydrophobic nature of its surface.

The observed peaks at 1022.6 and 1045.74 eV in the Zn2p spectrum shown in Fig. 3e correspond to the Zn 2p3/2 and Zn 2p1/2 in Zn–O, respectively^47^.

The C1S spectrum of the ZS@BIF sample is shown in Fig. 3f, in which the main core spectrum is divided into four multiplet peaks at 284.52, 285.22, 285.87, and 288.85 eV, which indicate the presence of C–H, C=C, and O=C–O/and or O–C–O functional groups of stearate^49–52^.

**Figure 4:**
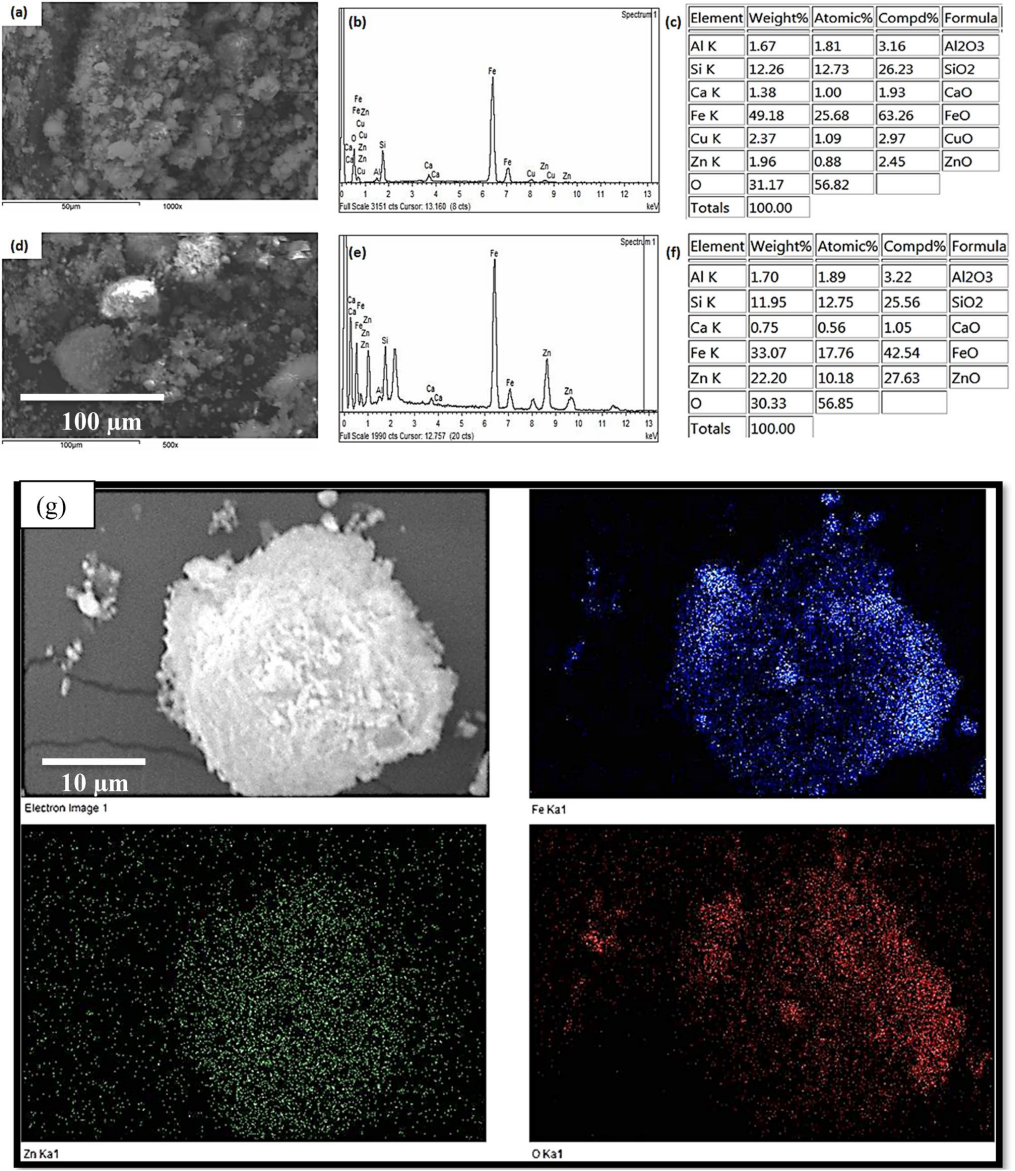
SEM, EDS spectra, and chemical analysis of (**a**–**c**) BIF and (**d**–**f**) ZS@BIF and EDS elemental mapping images for 20% ZS@BIF sample (**g**).

The SEM micrograph for BIF particles in Fig. 4a reveals quite spherical-like morphology with a grain size diameter of about 2–5 µm. High magnification SEM images are shown in Supplementary Figs. S2 and S3 (supplementary materials). Figure 4b,c shows the EDS spectrum and the corresponding elemental analysis of the BIF sample. The BIF sample contains about 49.18% Fe and 12.26% Si, which exactly matches the reported chemical analysis of BIF^35,36^.

On the other hand, the surface morphology of the 20% ZS@BIF sample displays spherical grains coated with very fine particles which have a cotton texture as shown in Fig. 4d. The EDS spectrum and elemental analysis of 20% ZS@BIF consist of 33.07% Fe, 11.95% Si, and 22.2% Zn as major constituents as depicted in Fig. 4e,f. Figure 4g represents the elemental mapping of the 20% ZS@BIF sample with different colors for the main elements. The observed image for Zn (green color) confirms the homogeneous distributions of zinc stearate over the particles of iron oxides which are characterized by blue and red colors for Fe and O respectively.

**Figure 5:**
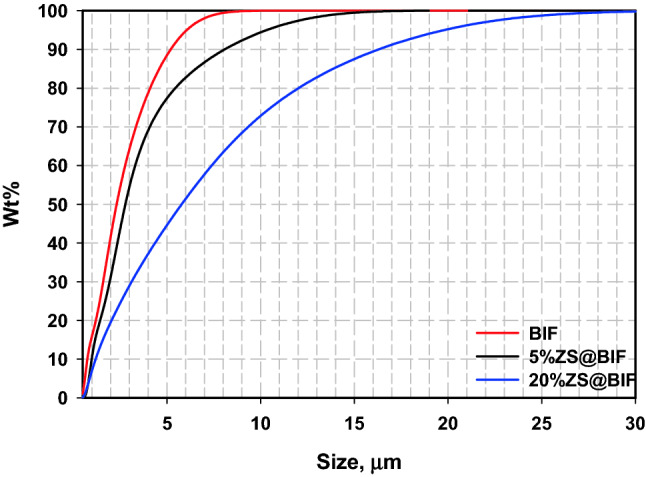
Particle size analysis of BIF, 5% ZS@BIF, and 20% ZS@BIF sample.

Particle size distribution of BIF, 5% ZS@BIF, and 20% ZS@BIF samples are displayed in Fig. 5. The dry coating process made significant changes in the BIF grain size distribution. Therefore, a slight shift in the average particle size (d_50_) was noticed. Apparently, the average particle size (d_50_) increased from 2.5 µm for the BIF to 2.8 µm for 5% ZS@BIF and 5.7 µm for 20% ZS@BIF. Likely, a dramatic increase was observed for the d_90_ value where it jumped from 5.1 to 8 µm and 16 µm for BIF, 5% ZS@BIF, and 20% ZS@BIF, correspondingly. The escalation in the grain size of the coated BIF samples confirms the formation of ZS@BIF composites.

Another indication for the particle size enlargement after the coating process could be observed from the results of density measurements as shown in Table 1. Coating the BIF with 5% zinc stearate could make a 10.4% decrease in the BIF density while a density decrease of 40.6% was obtained when the coverage percentage raised to 20%. The deviation of the measured densities from the calculated ones of ZS@BIF composites is attributed to the large numbers of voids (mainly air) attached to zinc striate because of its strong hydrophobic nature. These huge numbers of voids (30%) may enhance the capability of ZS@BIF composite to absorb a large amount of oily liquid.

**Table 1:** Density of BIF and ZS@BIF composites.

Sample	Density, g/cc		Density depression %	Voids%
	Measured	Calculated		
BIF	3.86	–	–	–
ZnS	1.10	–	–	–
5% ZS	3.46	3.73	10.39	7.28
20% ZS	2.29	3.31	40.61	30.68

**Figure 6:**
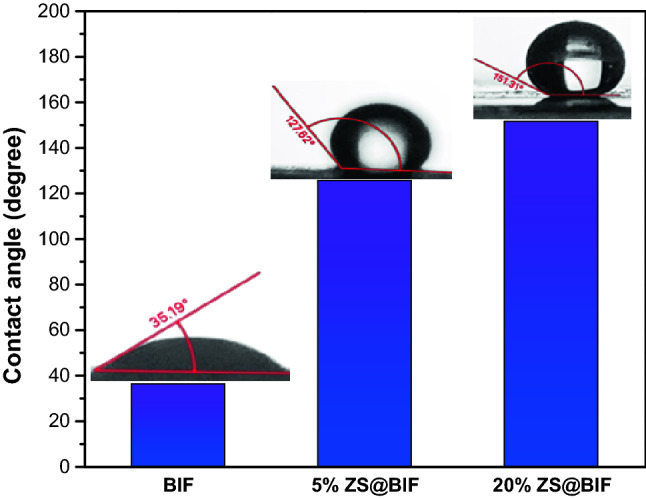
Contact angles with a water droplet on BIF, 5% ZS@BIF, and 20% ZS@BIF.

The contact angles increased from 35° to 127° and 151° by raising the percentage of ZS from 0 to 5% and 20% respectively as shown in Fig. 6. Thus, using zinc stearate as a coating layer was successful in producing a superhydrophobic composite for oil/water separation. The oil wettability was tested on these superhydrophobic composites, and it was found that the oil contact angle was 0° approximately indicating that both 5% and 20% ZS@BIF composite surfaces have a strong affinity to adsorb oil confirming that they are super oleophilic.

The magnetic susceptibility describes the degree of magnetization of the composites in response to an applied magnetizing field. It can be noted that the magnetic susceptibility for ZS@BIF composites gradually decreased with increasing ZS percent as shown in Supplementary Fig. S4 (supplementary materials). Although the addition of 20%ZS causes a noticeable reduction in the magnetic susceptibility of BIF, the produced 20%ZS@BIF still keeps a strong ferromagnetic character. Thus, the oil/composites phase could be effectively separated by conducting an external magnetic field.

### Performance evaluation

The adsorption capacities of superhydrophobic magnetic ZS@BIF composites for oil pollutants were studied to examine the oil/water separation performance as depicted in the recorded video V1 in the supplementary materials.

**Figure 7:**
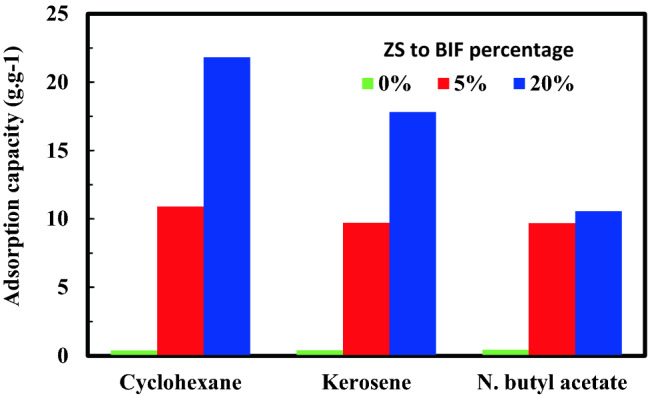
The adsorption capacity of oil on ZS@BIF treated with different percentages of ZS to BIF composite.

Figure 7 reveals the adsorption capacity of three different oil phases (cyclohexane, kerosene, and n. butyl acetate) per gram of BIF, 5% ZS@BIF, and 20% ZS@BIF composites. The adsorption capacity is highly related to the type of treated oil and the degree of hydrophobicity of the composites. 20% ZS@BIF exhibited the maximum adsorption capacity for cyclohexane of about 22 g g^−1^ (Fig. 7). This could be due to the very small relative polarity value of cyclohexane. The high efficiency of the ZS@BIF composites is mainly due to the high contact angle and strong magnetic capabilities.

Generally, the adsorption capacity of the newly developed 20% ZS@BIF composite exhibits moderate behavior in comparison with that of all magnetic hydrophobic composites previously reported in the literature for oil–water separation as described in Table 2.

**Table 2:** Comparison of the adsorption capacity for the currently developed sorbent with that of previously reported magnetic hydrophobic composites.

Magnetic sorbent	Sorption capacity (g/g)	Oil phase	References
PVDF/Fe_3_O_4_@polystyrene	36	Sunflower oil/diesel	^53^
Nanocelullose/oleic acid@Fe_3_O_4_	68	Cyclohexane	^54^
	56.3	Ethyl acetate	
	33.2	Vacuum pump oil	
Wood Sawdust@Fe_3_O_4_NPs/stearic acid	28.3	Motor oil	^55^
	41.2	Crude oil	
Polystyrene/Fe_3_O_4_/graphene aerogel	40	Crude oil	^56^
Magnetic carbon aerogel	10	Engine oil	^57^
	10.8	Corn oil	
Titanium tetraisopropoxide/Fe_3_O_4_ cellulose aerogel	28	Paraffin oil	^58^
Collagen/magnetite	2	Motor oil	^59^
Maghemite/polyurethane resin	10	Crude oil	^60^
Graphitic carbon/Fe_3_O_4_/polyurethane	34.2	Lubricating oil	^61^
CoFe_2_O_4_/sawdust	11	Lubricating oil	^62^
Surfactant capped magnetite	22.5	Petroleum oil	^5^
Zinc stearate/phenol formaldehyde-polyurethane sponge	62	Food oil	^28^
	81	Motor oil	
Zinc stearate/banded iron formation (ZS@BIF)	22.2	Cyclohexane	This work
	18	Kerosene	
	10.6	*N*-butyl acetate	

**Figure 8:**
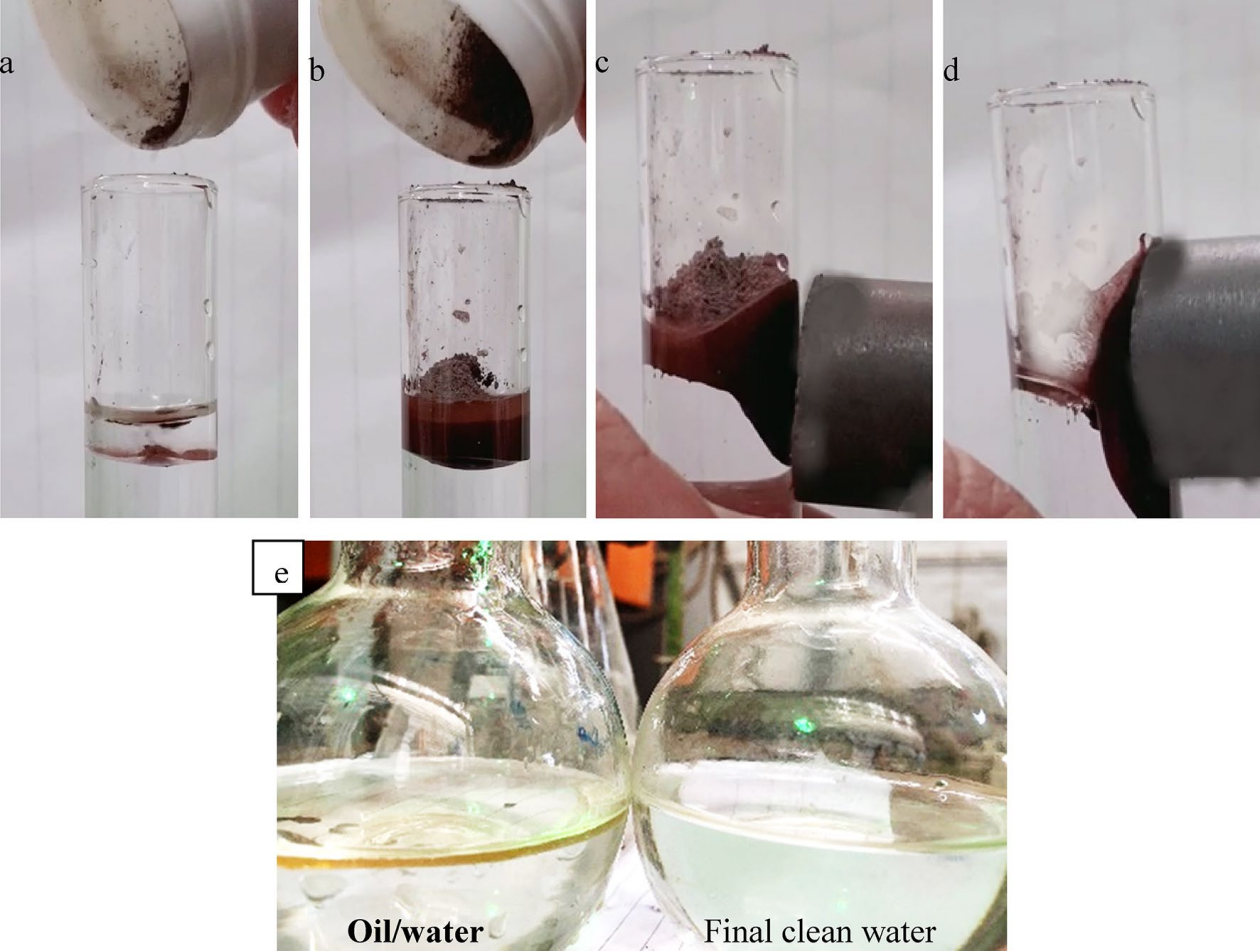
Oil/water separation using ZS@BIF composite (**a**) oil layer above water, (**b**) adding superhydrophoic magnetic composite, (**c**) magnet approaching the oil/composite layer, and (**d**) oil/composite layer attracted to an external magnetic field, (**e**) Image before and after oil/water separation using ZS@BIF composite.

It is worth mentioning that it is the first time to consider the magnetic nature of BIF ore to be employed as water-repellent and oil-adsorbing material as shown in Fig. 8a–d. Once the composite material was put in the oil/water mixture, oil was adsorbed quickly on the surface of composite particles forming the oil/particles layer. Then by applying an external magnetic field, the oil/particle layer was removed spontaneously to produce clean water. This test illustrated that the ZS@BIF composite material could be involved as a promising superhydrophobic/super oleophilic magnetic oil sorbent as shown in Fig. 8e.

**Figure 9:**
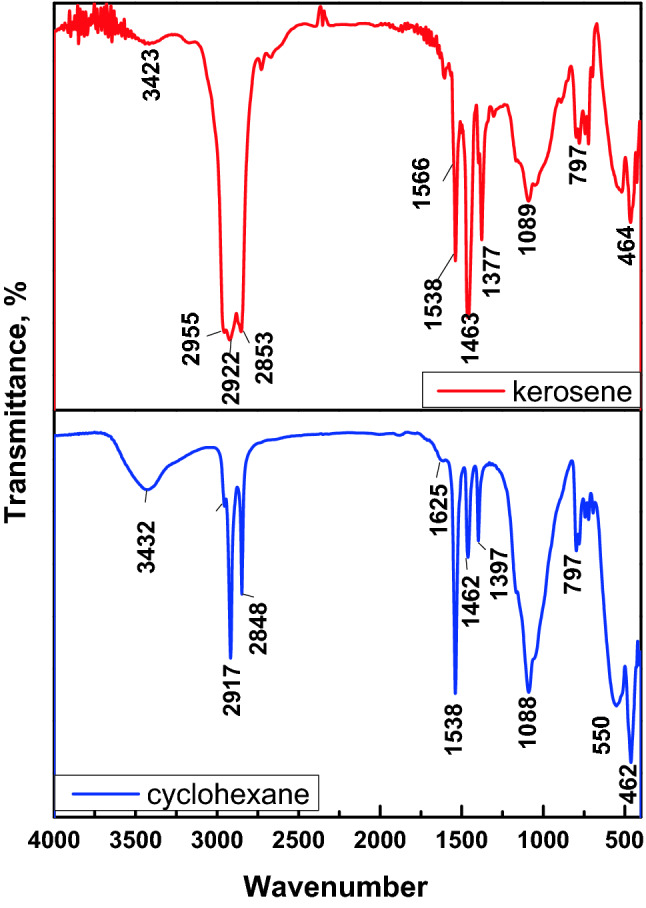
FTIR spectra of ZS@BIF composite loaded by kerosene and cyclohexane.

At the end of the oil/water separation experiments, the used hydrophobic magnetic sorbent ZS@BIF was collected by a hand magnet and after drying at room temperature, an FTIR investigation was conducted. As can be seen in Fig. 9, the characteristic peaks of cyclohexane, asymmetric and symmetric –CH_2_– stretching vibration bands at 2917 cm^−1^ and 2848 cm^−1^ respectively were observed^63^. The FTIR spectrum of ZS@BIF loaded with kerosene shows a typical shape of bands with pure kerosene. The sharp peaks observed at 2955 cm^−1^, 2922 cm^−1^, and 2853 cm^−1^ are ascribed to stretching the C–H alkanes group in kerosene^64^.

The amenability to recover the adsorbed oil (desorption) by rotary evaporator was examined as shown in the recorded Supplementary Video V2 (supplementary materials). First, the oil-loaded ZS@BIF was moved to the evaporating flask of the rotary evaporator, and then the temperature was set to + 10 °C over the boiling point of the studied oil for 15 min to achieve complete vaporization of the adsorbed oil and complete regeneration of the ZS@BIF sorbent. Results showed that for the studied oils, about 95% recoveries were achieved. Supplementary Fig. S5 in supplementary materials shows the oil-loaded ZS@BIF before and after the oil recovery experiment.

**Figure 10:**
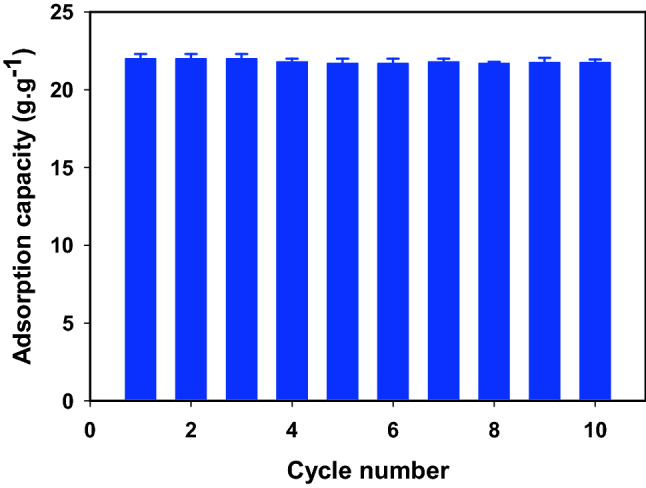
Adsorption performance of ZS@BIF composite for cyclohexane/water separation for 10 cycles.

To estimate the durability and reusability of the developed superhydrophobic magnetic composite, the regenerated ZS@BIF sorbent was tested for 9 repetitive cycles reuse. Figure 10 depicts the adsorption capacity of the fabricated ZS@BIF sorbent for cyclohexane against the number of reuse cycles. The developed ZS@BIF sorbent revealed excellent performance and high efficiency for oil adsorption without significant change up to 10 cycles. Therefore, this type of low-cost and eco-friendly processing technique could be considered an effective technique for the fabrication of several natural composites as efficient oil adsorbents.

## Conclusions

Natural superhydrophobic magnetic sorbent (ZS@BIF) was successfully prepared via a one-step, low-cost, and simple hybridization technique. The structural investigation using XRD, SEM, and EDS confirmed the formation of the ZS@BIF composite. Elemental mapping of the ZS@BIF composite sample revealed the excellent and uniform dispersion of zinc stearate between the iron oxide particles. Surface wettability studies indicated that the water contact angles significantly increased from 35° for un-treated BIF to 151° for 20%ZS@BIF composite suggesting superior hydrophobicity for the fabricated novel magnetic sorbent. The prepared ZS@BIF showed the highest affinity to adsorb cyclohexane than n-butyl acetate and kerosene with a maximum adsorption capacity of about 22 g g^−1^. The fabricated ZS@BIF composite revealed an excellent performance with 99.9% removal efficiency and high recyclability over ten repeated cycles.

## Supplementary Information

Supplementary Figures.

Supplementary Video 1.

Supplementary Video 2.

## Data Availability

The datasets generated and/or analysed during the current study are not publicly available due to institutional roles and confidential conditions but are available from the corresponding author on reasonable request.
