# Supplementary material for: Superhydrophobic magnetic sorbent via surface modification of banded iron formation for oily water treatment
Source: Sci Rep. 2022 Jun 30;12:11016. doi: 10.1038/s41598-022-15187-6 (PMC9246911; doi:10.1038/s41598-022-15187-6)
Supplement: Supplementary file 1 — Supplementary Figures. [file 41598_2022_15187_MOESM1_ESM.docx]

**Superhydrophobic magnetic sorbent via surface modification of banded iron formation for oily water treatment**

**Mohsen Farahat^*^, Ahmed Sobhy^*^, Moustafa M.S. Sanad^*^**

*Central Metallurgical Research and Development Institute, Helwan, Cairo 11421, Egypt.*


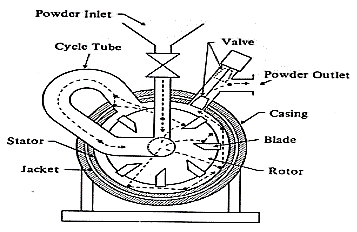


Fig.S1: Schematic illustration of the Hybrizier (Bhaumik, 2015)


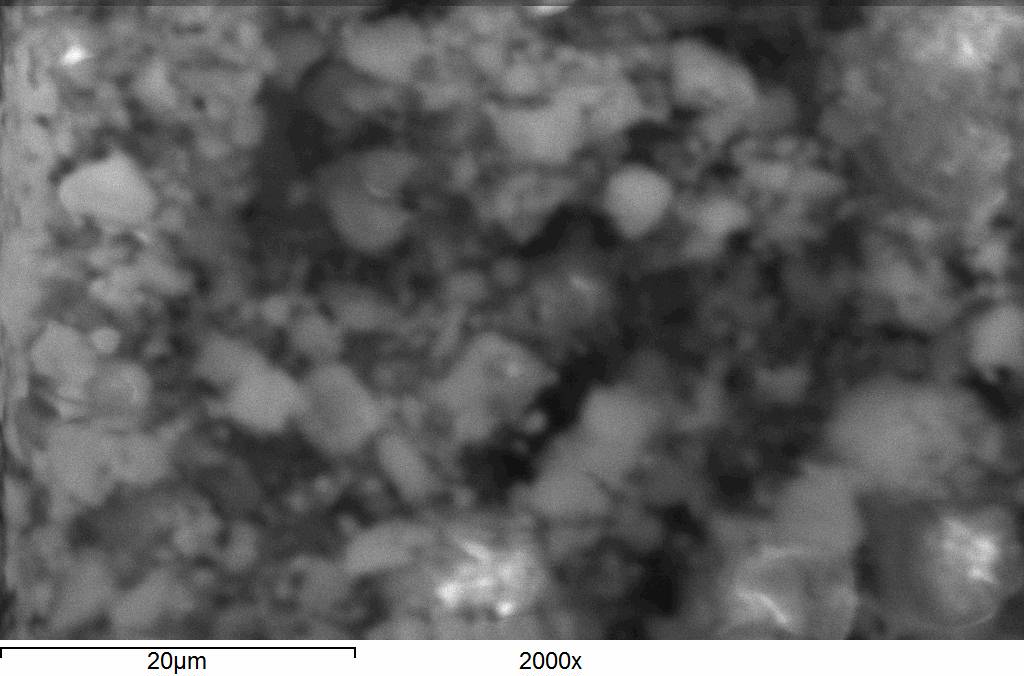


Fig. S2: SEM of BIF with higher magnification


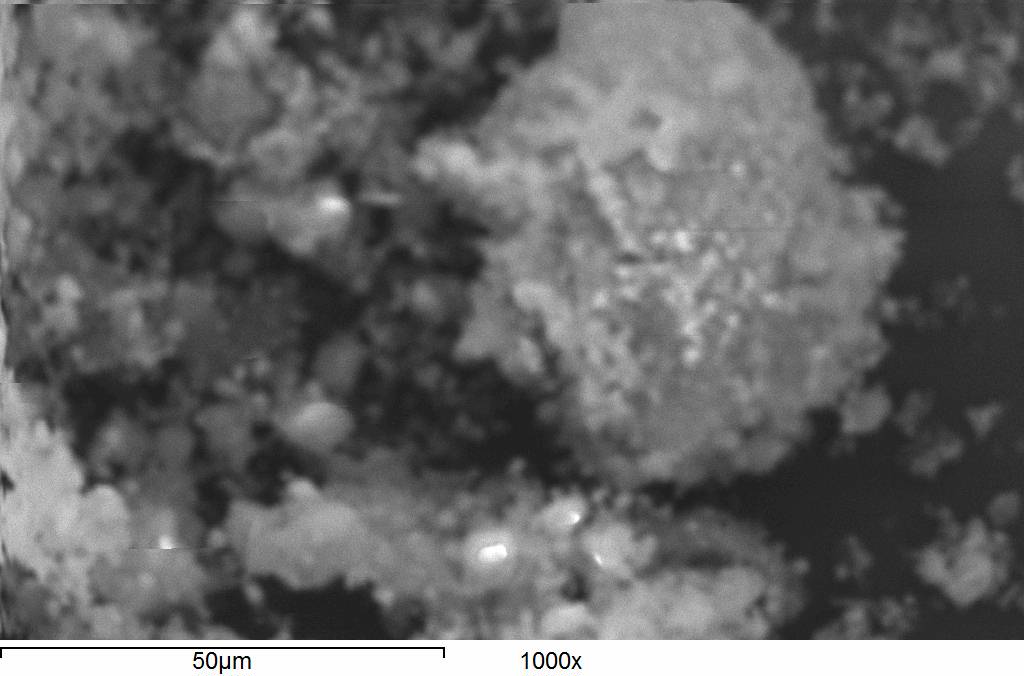


Fig. S3: SEM of ZS@BIF with higher magnification

Fig.S4. Magnetic susceptibility of BIF, 5%ZnSt@BIF, and 20% ZnSt@BIF samples


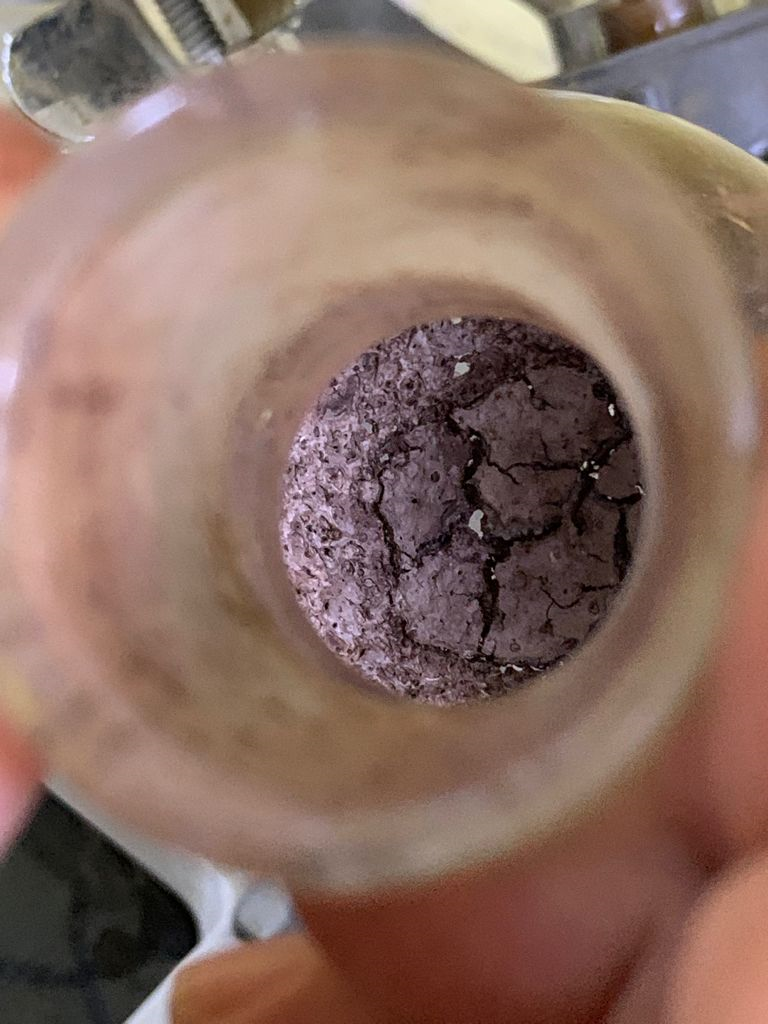

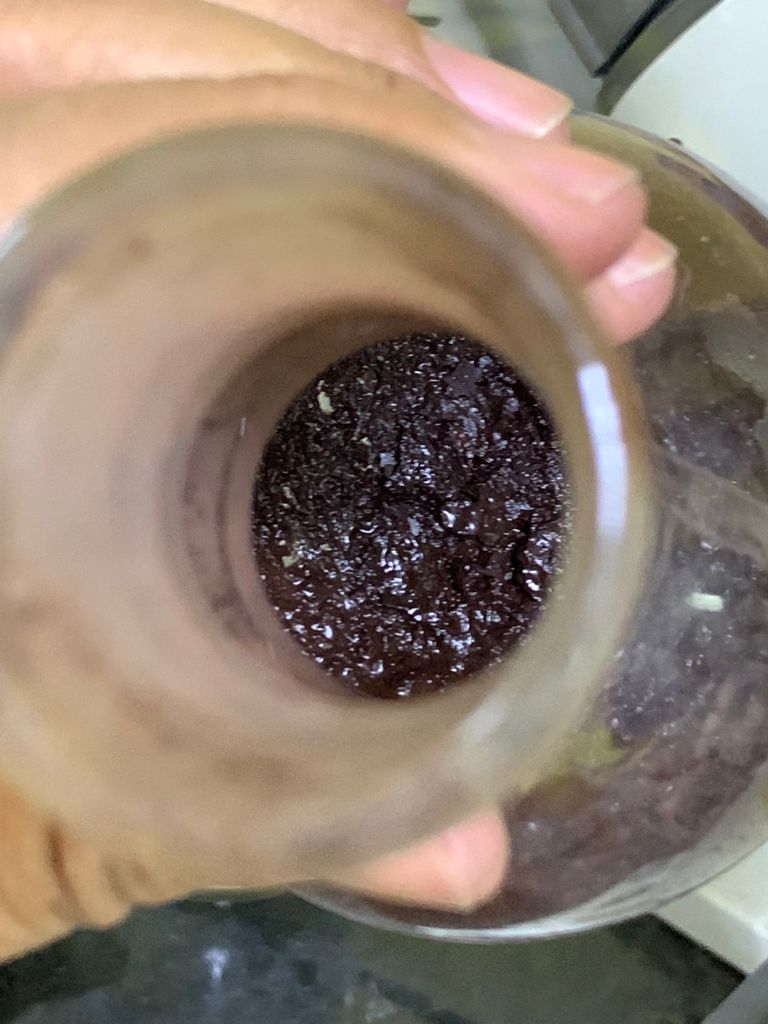


Fig.S5: Oil-loaded ZS@BIF before (left) and after (right) oil recovery via rotary evaporator
